# The cost-effectiveness of different visual acuity screening strategies in three European countries: A microsimulation study

**DOI:** 10.1016/j.pmedr.2022.101868

**Published:** 2022-06-27

**Authors:** Eveline A.M. Heijnsdijk, Mirjam L. Verkleij, Jill Carlton, Anna M. Horwood, Maria Fronius, Jan Kik, Frea Sloot, Cristina Vladutiu, Huibert J. Simonsz, Harry J. de Koning, for the EUS€REEN Foundation

**Affiliations:** a Department of Public Health, Erasmus University Medical Center, Rotterdam, The Netherlands; b School of Health and Related Research (ScHARR), University of Sheffield, Sheffield, United Kingdom; c Infant Vision Laboratory, School of Psychology and Clinical Language Sciences, University of Reading, Reading, United Kingdom; d Goethe University, Department of Ophthalmology, Child Vision Research Unit, Frankfurt am Main, Germany; e Department of Ophthalmology, Erasmus University Medical Center, Rotterdam, The Netherlands; f University of Medicine and Pharmacy, Cluj-Napoca, Romania

**Keywords:** Cost-effectiveness, Visual acuity, Screening, QALYs, quality adjusted life-years, VA, visual acuity, MISCAN, MIcrosimulation SCreening Analysis, ICER, incremental cost-effectiveness

## Abstract

Childhood vision screening programmes in Europe differ by age, frequency and location at which the child is screened, and by the professional who performs the test. The aim of this study is to compare the cost-effectiveness for three countries with different health care structures.

We developed a microsimulation model of amblyopia. The natural history parameters were calibrated to a Dutch observational study. Sensitivity, specificity, attendance, lost to follow-up and costs in the three countries were based on the EUSCREEN Survey. Quality adjusted life-years (QALYs) were calculated using assumed utility loss for unilateral persistent amblyopia (1%) and bilateral visual impairment (8%). We calculated the cost-effectiveness of screening (with 3.5% annual discount) by visual acuity measurement at age 5 years or 4 and 5 years in the Netherlands by nurses in child healthcare centres, in England and Wales by orthoptists in schools and in Romania by urban kindergarten nurses. We compared screening at various ages and with various frequencies.

Assuming an amblyopia prevalence of 36 per 1,000 children, the model predicted that 7.2 cases of persistent amblyopia were prevented in the Netherlands, 6.6 in England and Wales and 4.5 in Romania. The cost-effectiveness was €24,159, €19,981 and €23,589, per QALY gained respectively, compared with no screening. Costs/QALY was influenced most by assumed utility loss of unilateral persistent amblyopia. For all three countries, screening at age 5, or age 4 and 5 years were optimal.

Despite differences in health care structure, vision screening by visual acuity measurement seemed cost-effective in all three countries.

## 1 Introduction

Amblyopia (‘lazy eye’) is a mostly unilateral decrease of visual acuity (VA), which develops in early childhood. It is caused by refractive error and/or strabismus (misalignment of the eyes), or by stimulus deprivation (such as congenital cataract). The prevalence of amblyopia ranges between 2% and 4%, depending on the definition of amblyopia used and the population (Solebo et al., 2015).

The impact of unilateral vision loss caused by persistent amblyopia (either detected too late or unsuccessfully treated) is probably small, but remains for the entire life (Chua and Mitchell, 2004, Rahi et al., 2006). Amblyopia can also lead to bilateral visual impairment if the function of the non-amblyopic eye is affected by disease or trauma in older age. Children aged six years with persistent amblyopia spend, on average, the last 15 months of their life with bilateral visual impairment (decimal VA < 0.5, or logMAR worse than 0.3) against 8 months for healthy six-year-olds (van Leeuwen et al., 2007). Due to the maturing nature of the visual system, treatment of amblyopia should preferably start before age 6–7 years to be optimally effective (Holmes et al., 2011, Flynn et al., 1998). Screening can contribute in timely detection of amblyopia and, if followed by successful treatment, can prevent persistent amblyopia.

Although the costs of a vision screening programme and the costs per amblyopia case detected are low, vision screening might not be cost-effective (van Leeuwen et al., 2007, van de Graaf et al., 2010). This is due to the small impact amblyopia has on quality of life and that the benefit of avoiding bilateral visual impairment emerges many years after screening and treatment of amblyopia have taken place. Only a few studies estimated the cost-effectiveness using costs per quality adjusted life-year (QALY) over a life-time horizon (Carlton et al., 2008, Konig and Barry, 2004, Rein et al., 2012, Asare et al., 2022). These studies concluded that the assumed utility loss of unilateral visual impairment had a large impact on the costs per QALY. When zero utility loss of unilateral visual impairment was assumed, screening was not cost-effective.

Screening for amblyopia is widely implemented in most countries in Europe, but screening programmes are diverse in age, test, frequency of testing, and setting (Sloot et al., 2015). By calculation of the cost-effectiveness and determination of the optimal programme, this diversity might be reduced.

Different national or regional circumstances might require different screening strategies, especially to maximise the attendance rate and reduce overhead costs. Depending on the existing structures in a country, vision screening programmes may be most efficiently organized at kindergartens, (pre)school or combined with general pediatric examinations or vaccinations. For example, in the Netherlands all children are invited for health checks (including vision screening) at child healthcare centers. VA screening is performed twice on average at ages 45 and 63 months by youth healthcare nurses or doctors (Mazzone et al., 2018, de Koning et al., 2013). In England and Wales the recommendation (The UK NSC recommendation on Vision defects screening in children) is to screen VA in school by an orthoptist-led service once between age 4–5 years (Mazzone et al., 2018, Yau et al., 2020). In Romania, vision screening between age 4 and 5 years was implemented in the county of Cluj in 2018 and 2019 as part of the EUSCREEN Study (Kik et al., 2021). In the cities, vision screening was done by kindergarten nurses. In the rural areas, screening had less coverage, since the kindergartens are smaller and there are no resident nurses. We will focus this paper on the urban areas with good coverage.

The aim of this study is to determine whether vision screening is cost-effective. We developed a microsimulation model to compare the cost-effectiveness of the current and alternative screening strategies in three countries: The Netherlands, England and Wales, and Romania, chosen because they have different health care structures, are part of the EUSCREEN study and provided data (Bussé et al., 2021) to use in the model.

## 2 Methods

### 2.1 Microsimulation SCreening ANalysis (MISCAN) model

**Fig. 1:**
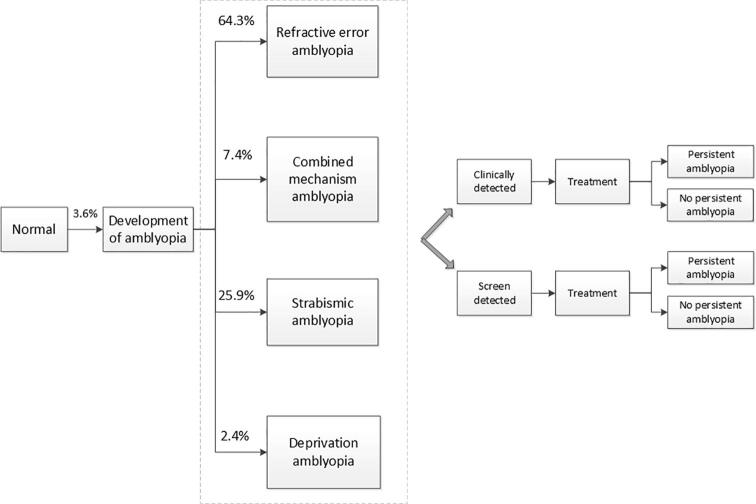
The MISCAN-Vision model structure. General MISCAN-Vision model structure including the natural history of amblyopia prior to diagnosis by either clinical detection or screening. Preclinical transition probabilities are indicated next to the arrows. The model has been calibrated to the results of the Optimisation of Amblyopia Screening (OVAS) study with 10,811 children and 344 cases of amblyopia. (Sloot et al., 2022)The proportion of refractive error amblyopia is higher than in a previous study, (de Koning et al., 2013)partly because refractive error amblyopia that was corrected by glasses at a follow-up examination was not included.

We developed a stochastic micro-simulation model using the MIcrosimulation SCreening ANalysis (MISCAN) model, extensively used for evaluating cancer screening (Heijnsdijk et al., 2012, Sankatsing et al., 2015). The model simulates the natural history of a disease and simulates repeated screens at various ages. The model follows the individual life events from birth to death (Fig. 1). All children are born with absence of amblyopia (Hubel and Wiesel, 1963), then a proportion of children develop amblyopia in the first years of life. In case of development of amblyopia, the child progresses into one of the four model states: preclinical (undetected) amblyopia caused by refractive error, by strabismus, by a combination of these or by deprivation. These preclinical amblyopic cases remain undetected until detection by either screening or clinical consultation after concerns by the parents. Detection is followed by one of the possible treatments: glasses, patches to occlude the healthy eye, a combination of these treatments. Depending on age, there is a probability that treatment is successful and that the child reaches sufficient VA to read with the amblyopic eye (at least decimal VA 0.5 or 0.3 logMAR) (Flynn et al., 1998). Unsuccessful treatment or failure to timely detect amblyopia results in persistent amblyopia.

Model parameters were based on literature, expert opinion and calibration. For the calibration we used data from the Optimisation of Amblyopia Screening study (OVAS) (Sloot et al., 2022). In this Dutch study, two consecutive birth cohorts were followed. All results of all vision screening episodes during general health examinations were collected. In the control group (standard strategy at that moment), 5,649 children were screened on average 3.1 times with orthoptic tests performed by child healthcare doctors or nurses, between age 6 and 24 months, followed by VA screening at age 36 and 45 months. In the intervention group consisting of 5,162 children, the screens between age 6 and 24 months were only performed in case of visually apparent abnormalities or family history.

The result of the VA measurement could be insufficient (when the threshold was not reached) or fail (when it was not possible to determine VA). Failed tests could be repeated. The amblyopia diagnosis was based on the first VA measurement by the orthoptist to whom the child was referred. This classification was mainly based on a VA difference of 2 logMAR lines between the eyes or a bilateral ≤ 0.5 Snellen VA (at age 4 years) before the start of treatment, but with spectacle correction. In total 344 cases of amblyopia were found, 298 of which by screening.

### 2.2 Development of the vision screening model

We assumed that amblyopia develops at earliest at three months after birth (Hubel and Wiesel, 1963). In addition, we assumed that amblyopia caused by refractive error cannot be detected before age 3 years as VA measurement before this age is not accurate and photoscreening is not detecting amblyopia. Using the data of the control arm of the OVAS study, we calibrated the parameters for the cumulative incidence of amblyopia by age, the distribution of the four amblyopia types, the duration of the preclinical states and the screening test sensitivities by age and amblyopia type. The Nelder and Mead optimization method (Barton and Ivey, 1996) was used to minimize the difference between the predicted number of cases of amblyopia by age, type and detection (by screening or clinical consultation) with the observed numbers in the OVAS study. The calibrated model was validated using the intervention arm of the OVAS study (Supplementary Figs. 1–5 and Supplementary Table 1).

Treatment started at age 5 to 7 years is less effective, i.e. longer patching is needed and the success declines accordingly (Holmes et al., 2011, Fronius et al., 2014). We assumed that the probability of successful treatment is 75% (Flynn et al., 1998, Konig and Barry, 2004) at the age of three months to 5.5 years and then declines linearly until it reaches 5% at age twelve years.

### 2.3 Quality of life

Children with unsuccessful detection or treatment in the model progress to persistent amblyopia and therefore unilateral visual impairment for their remaining life. In a cohort study it was found that children with persistent amblyopia have bilateral visual impairment (VA < 0.5), on average the last 15 months of their lives, compared with 8 months for children without amblyopia or with successfully treated amblyopia (van Leeuwen et al., 2007). Using the time trade-off method (asking how many years people want to exchange for perfect health), the loss in utility measured in elderly people (mean age 75 years) with bilateral visual impairment was 0.08 (8% loss in quality of life) (van de Graaf et al., 2016). Using the same methods, the effect of unilateral visual impairment measured in 35–40-year olds was 0.037 (3.7% loss in quality of life) (van de Graaf et al., 2010). However, in the same study the standard gamble method, in which a person accepts a very small risk of dying, indicated that only 37% of the people with unilateral visual impairment accepted a death risk. Since the 0.037 utility loss for unilateral visual impairment is considered high compared to the 0.08 utility loss for bilateral visual impairment, we assumed a loss in utility for unilateral visual impairment of 0.01, which has been used before. (Rein et al., 2012) In the base analysis, we assumed no utility loss for the treatment of amblyopia.

### 2.4 Model runs using country-specific parameters

For each country separately, the current protocol was simulated and compared with 15 alternatives Country-and age-specific attendance to screening, percentage referral and percentage of compliance with referral were used (Table 1). We also used country-specific life tables, distributions of treatment and costs of screening (varying by age), diagnosis and treatment for the year 2018. These costs were obtained from expert opinion and converted to Euros based on the current exchange rate.

**Table 1:** The country-specific input parameters in the MISCAN model.

	The Netherlands	England and Wales	Romania
Current screening ages	45 (42–48) and 60 (54–66) months	4 or 5 years	4 or 5 years
Screening professional	youth doctor or youth nurse	Orthoptist, or school nurse, healthcare assistant, orthoptic assistant managed by orthoptist	nurse
% attendance to screening (of all eligible children)	36 months: 90% 45 months: 89% 60 months: 84%	92% (Yau et al., 2020)	75% (Kik et al., 2021)
% repeat screen	36 months: 20% (Telleman et al., 2019) 45 months: 11% (Vision in Preschoolers Study Group et al., 2006) 60 months: 7% (Vision in Preschoolers Study Group et al., 2006)	3.5% (Horwood et al., 2021)	2.4% (Kik et al., 2021)
% referral after screening	36 months: 4.6% (Sloot et al., 2022) 45 months: 5.5% (Sloot et al., 2022) 60 months: 4%	13% (Yau et al., 2020)	12.5% (Kik et al., 2021)
% compliance to diagnostics	69% (Sloot et al., 2022)	76% (Horwood et al., 2021)	50%, assumption
Number of unscreened children visiting an ophthalmologist to detect one case	2 (Sloot et al., 2022)	Assumption: same as in the Netherlands	Assumption: same as in the Netherlands
Treatment distribution - Glasses - Patching - Combined	15% (de Koning et al., 2013) 12% 73%	Assumption: same as in the Netherlands	Assumption: same as in the Netherlands
Costs of screening	36 months: € 2.5 ≥36 months: € 2	£ 4.22 (Horwood et al., 2021)*	€ 3.17
Costs of diagnosis	€ 150	£ 190 (Horwood et al., 2021)*	€ 25
Costs of treatment: - Glasses - Patching - Combined	€ 2000 € 2500 € 3000	£ 1142 (Horwood et al., 2021)* £ 1096 £ 1192	€ 2000
Costs of bilateral visual impairment	€ 1000 per year assumption	€ 1000 per year assumption	€ 1000 per year assumption

*an exchange rate of 1.18 of Euros to English Pounds was used.

In the Netherlands, screening is performed in combination with health checks and vaccination, therefore the attendance is high (84–95%). (Mazzone et al., 2018) The percentage repeated screens (7–20%) and referral (4–5.5%) per age is known. To account for diagnostic activity for children not referred by screening, we assumed that 2–3 referred children will have a diagnostic examination to detect one child with amblyopia (Sloot et al., 2022, Sloot et al., 2015).

Since the screeners in England and Wales are orthoptists or trained by orthoptists, we assumed a 10% higher test sensitivity compared to the screening by youth healthcare nurses in the Netherlands. The percentage repeated screens (3.5%) (Horwood et al., 2021) and referral (13%) (Yau et al., 2020) are known for ages 4 to 5 years. The assumptions for diagnostic activity and treatment were the same as for the Netherlands.

For screening in Romania by urban kindergarten nurses, we assumed a 10% lower test sensitivity as they only started recently (January 2018). For ages 4 to 5 years, 2.4% of the screens were repeated and the referral was 12.5% (Kik et al., 2021). Based on the limited amount of ophthalmologists and the lack of orthoptists in Romania, we accounted for a lower clinical detection probability in Romania by increasing the durations of the preclinical stages with 20%.

Next, for each country, 15 alternative screening strategies with measurement of VA, varying in age (3–6 years) and screening frequency (0–4 times) were simulated, assuming the same attendance, referral and compliance to referral as in the nearest age categories, when these values are unknown. Screens could take place during the whole year at the eligible age.

To reduce random noise, we simulated 10 million children followed over life-time and scaled the results to 1,000 children. An annual 3.5% discount (recommendation in the National Institute for Health and Care Excellence (NICE) guideline) for costs as well as QALYs was used to convert future costs and health effects to the present (the yearly discount reflects the yearly interest of state bonds, because the government has to borrow money to invest in new interventions). For each strategy, model outcomes were number of screens, referrals, cases detected, cases of persistent amblyopia, life-years with unilateral and bilateral visual impairment, costs and QALYs gained. The efficiency frontier consists of all strategies gaining the most QALYs against the least costs. We calculated the incremental cost-effectiveness (ICER) of the strategies by dividing the additional costs by the additional QALYs gained compared with the nearest, less expensive strategy on the cost-effectiveness frontier. Cost-effectiveness was analyzed from a modified health care perspective, with the consequences that societal costs (e.g. travel costs for the parents) and benefits (e.g. increased work productivity) were not included. Assumed costs of support of elderly with bilateral visual impairment due to persistent amblyopia (€1,000 per year) are included.

One-way sensitivity analyses were performed for variation in attendance (80% and 100%), utility loss of treatment (0.01 for 1 year), utility loss for bilateral visual impairment (0.22) (Konig and Barry, 2004, Sharma et al., 2000), success of treatment of amblyopia (85% and 65% until age 5.5), screening test sensitivity and referral (10% lower and higher than base values), and costs (50% lower and higher). In addition we varied the utility loss for unilateral visual impairment. The lower value was set at 0.005, in contrast of two studies that used 0 (Carlton et al., 2008, Konig and Barry, 2004). We used 0.005 since it is unlikely that quality of life is not affected at all. The upper value was 0.02, as has been used previously (Carlton et al., 2008, Rein et al., 2012).

## 3 Results

Without screening, 36 children in a population of 1,000 children are estimated to develop amblyopia. The model predicted that in the Netherlands in a situation without screening in these 1,000 children 73.4 will be referred to diagnostic assessment and 17.7 will have persistent (undetected or unsuccessfully treated) amblyopia at age 6 years and beyond (Table 2), resulting in a loss of 12.55 QALYs due to unilateral and 0.54 QALYs due to bilateral visual impairment.

**Table 2:** The effects and costs of the current screening programme of The Netherlands, England and Wales and Romania, compared to their situation without screening. All results are presented per 1,000 children, followed over life-time.

	**The Netherlands**			**England and Wales**			**Romania**		
	No screen	Screen	Difference	No screen	Screen	Difference	No screen	Screen	Difference
**No discount**									
Screens (including repeated screens)	0	1,846	1,846	0	917	917	0	759	759
Referrals	73.4	108.9	33.7	77.6	153.4	75.8	76.3	140.0	63.7
Cases detected by screening	0	24.8	24.8	0	21.7	21.7	0	14.5	14.5
Persistent amblyopia	17.7	10.4	−7.3	17.7	11.1	−6.6	18.6	14.1	−4.5
Life-years with persistent amblyopia	1,262	759	−503	1,250	803	−448	1,215	938	−277
Life-years with bilateral visual impairment	13.6	7.9	−5.7	13.3	8.3	−4.9	11.9	9.0	−2.9
QALYs lost due to unilateral visual impairment	12.55	7.55	−5.00	12.44	7.99	−4.45	12.09	9.33	−2.76
QALYs lost due to bilateral visual impairment	0.54	0.32	−0.23	0.53	0.33	−0.20	0.47	0.36	−0.12
**With 3.5% discount**									
QALYs lost due to unilateral visual impairment	3.19	2.01	−1.19	3.18	2.12	−1.06	3.13	2.48	−0.64
QALYs lost due to bilateral visual impairment	0.04	0.02	−0.02	0.04	0.02	−0.01	0.03	0.03	−0.01
QALYs gained	0	1.20	1.20	0	1.07	1.07	0	0.65	0.65
Costs of screening	0	3,117	3,117	0	2,309	2,309	0	2,027	2,027
Costs of diagnosis	8,348	13,894	5,547	8,345	19,336	10,992	1,305	2,846	1,540
Costs of treatment	63,209	83,781	20,571	30,323	38,612	8,288	36,844	48,765	11,921
Costs bilateral visual impairment	484	283	−201	472	296	−176	422	320	−102
Total costs	72,041	101,075	29,033	39,140	60,553	21,413	38,571	53,958	15,387
Costs/QALY gained			24,159			19,981			23,589

**Fig. 2:**
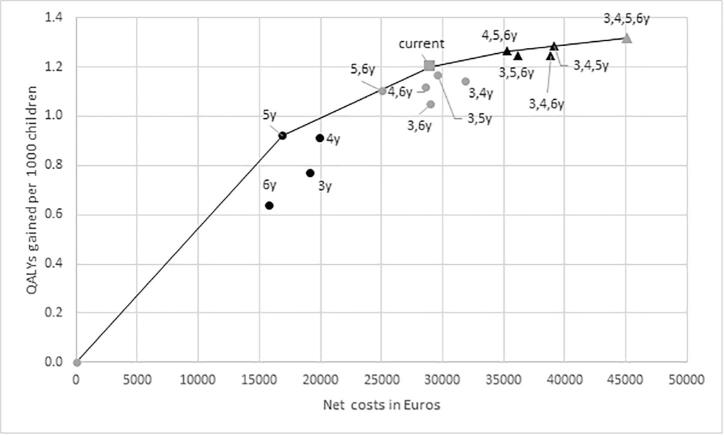
Net costs and QALYs gained. The net costs and QALYs gained of the current screening programme in the Netherlands and alternative screening programmes. Costs and QALYs are discounted with 3.5%. The black dots are the programmes with one screen, the grey dots with 2 screens, the black triangles with 3 screens and the grey triangle with 4 screens. The grey square is the current Dutch programme, with screens at age 42–48 months and 54–66 months. The black line is the efficiency frontier: strategies on this line result in the highest QALYs against the lowest costs and are therefore optimal.

The current Dutch screening programme resulted in 1,846 screens, 35.5 more referrals and 7.3 cases of persistent amblyopia prevented (a reduction of 41%) compared with no screening. Less QALYs were lost: 7.55 due to unilateral, and 0.32 due to bilateral visual impairment. Using a 3.5% discount rate, screening resulted in 1.19 QALYs gained for unilateral and 0.02 QALYs gained for bilateral visual impairment. The costs per QALY gained were €24,159 compared with no screening. With a birth number of 170,000 each year, about 1,241 cases of persistent amblyopia will be prevented. The costs and QALYs of all hypothetical strategies are presented in Fig. 2. Strategies consisting of one or two screens were less expensive, but also gained less QALYs. Strategies on the cost-effectiveness frontier were screening at age 5 years (ICER €18,399, compared with no screening), screening at age 4 and 5 years (ICER €42,952, compared with age 5), screening at age 4, 5 and 6 (ICER €100,151, compared with age 4 and 5) and screening at age 3, 4, 5 and 6 years (ICER €188,774, compared with age 4, 5 and 6) (Supplementary Tables 2 and 3).

**Fig. 3:**
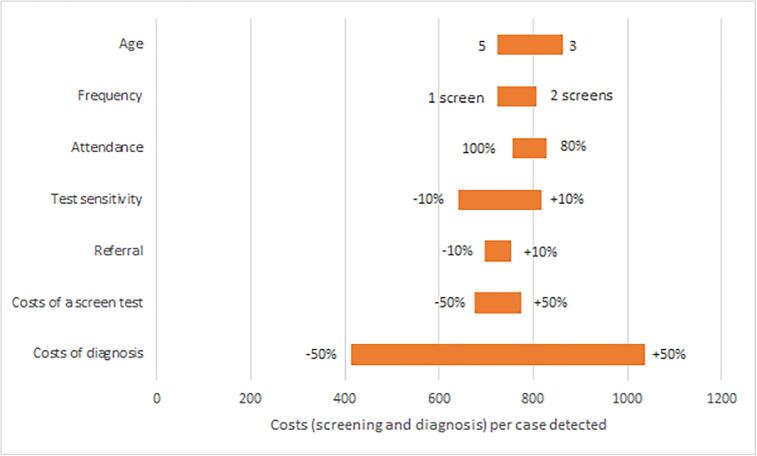
Costs per case detected by screening. Sensitivity analysis for the costs (of screening and diagnosis) in Euros per case detected by screening. One screen at age 5 years was used as the base model. The alternative parameter values (low and high value) are indicated next to the bars. In that strategy the costs per case detected were €724.

**Fig. 4:**
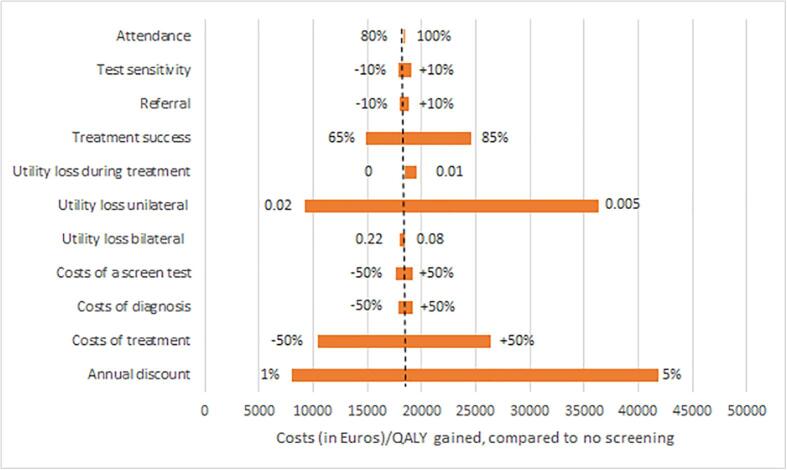
Costs per QALY gained for one screen at age 5. Results of the univariate sensitivity analysis: the costs/QALY gained for one screen at age 5 in the Netherlands using alternative parameter values. The parameter values (low and high value) are indicated next to the bars. The costs/QALY gained for the base model was €18,399 (dashed line). All sensitivity analyses were calculated with an annual discount of 3.5%, except for the last bar, in which the discount was varied between 1% and 5%.

The costs of screening and diagnosis per case detected were estimated to be €724 for one screen at age 5, and €807 for screens at age 4 and 5 years. The test sensitivity and costs of diagnosis also had a large impact on the costs per case detected (Fig. 3). The sensitivity analysis for cost-effectiveness showed that the results strongly depended on the assumed utility loss for unilateral visual impairment (Fig. 4). Assuming 0.005 or 0.02 utility loss for unilateral visual impairment resulted in ICERs of €36,309 and €9,262 for screening at age 5 years compared with no screening. The discount rate also had a large impact. Assuming 50% less or increased treatment costs would lead to ICERs of €10,415, and €26,382 per QALY gained respectively. A lower success of treatment (65%) resulted in an ICER of €14,858, whereas 85% treatment success resulted in an ICER of €24,577. All remaining parameters had minor effects. For most parameter values, the cost-effectiveness of screening at age 5 years was below €20,000 and the same strategies (age 5, age 4 and 5, age 4, 5 and 6, and age 3, 4, 5, 6 years) were on the cost-effective frontier (Supplementary Table 4).

The screening programme in England and Wales, consisting of one VA measurement between age 4 to 5 years at school entry, resulted in 917 screens, 75.8 additional referrals, 6.6 cases of persistent amblyopia prevented (37% less than without screening) and a discounted 1.07 QALYs gained per 1,000 children compared with no screening (Table 2). The costs per QALY gained were €19,981. With 640,000 births each year, about 4,224 cases of persistent amblyopia will be prevented. The costs and QALYs of alternative screening strategies are presented in Supplementary Fig. 6 and Supplementary Table 5. The costs and QALYs of the current programme are close to the cost-effective strategy of one screen at age 5 years (ICER €19,118).

The screening programme that was implemented in the urban areas of Cluj used VA measurements at age 4 or 5 years. The model predicted that screening in urban areas resulted in 759 screens, 63.7 additional referrals, 4.5 cases of persistent amblyopia prevented (24% less than without screening) and 0.65 discounted QALYs gained per 1,000 children compared with no screening (Table 2). The costs per QALY gained were €23,589. If this programme would be applied nationally (178,000 births each year), about 800 cases of persistent amblyopia will be prevented. Screening at age 5 years (1 screen), age 4 and 5 years (2 screens), at age 3, 4, and 5 years (3 screens) and at 3, 4, 5 and 6 years (4 screens) were on the efficiency frontier (Supplementary Fig. 7 and Supplementary Table 6).

## 4 Discussion

We found that although the countries have different protocols and organization of VA screening, the cost-effectiveness was comparable, around €20,000. For the United Kingdom this is below the NICE threshold of £20,000–30,000 (€23,496–€35,243). In the Netherlands and Romania no formal thresholds are used, but the cost-effectiveness is below the threshold of 1 to 3 times the GDP per capita as the WHO suggests (Cost effectiveness and strategic planning) (US$59,229 (€50,345) in the Netherlands and US$31,946 (€27,154) in Romania). For Romania, we modeled screening in urban regions, however almost half of the Romanian population lives in rural areas. In rural areas, kindergartens are smaller and there are no resident nurses (Kik et al., 2021). Therefore, screening will be less cost-effective in rural areas.

The cost-effectiveness results strongly depended on the assumed utility loss for unilateral visual impairment. Three earlier studies demonstrated that screening was much less cost-effective when using a utility loss of 0 or 0.01 for unilateral visual impairment, as compared to 0.02 or 0.04 (Carlton et al., 2008, Konig and Barry, 2004, Rein et al., 2012). Given this large impact, more research is needed to better estimate the impact of unilateral visual impairment on quality of life (Asare et al., 2022, Nilsson, 2007). Currently, only one study is available, reporting a high utility loss of 3.7% among 35–40 years old people (van de Graaf et al., 2010). The impacts of bilateral visual impairment or utility loss because of treatment on the QALYs gained in the population were much smaller, because of the shorter period of these states.

The model predicted that age 5 years was the optimal age for a single screen programme. Most cases of amblyopia will have developed by age 5 years and the testability of a child is higher than at age 3 years (Kvarnström and Jakobsson, 2005, Telleman et al., 2019, Vision in Preschoolers Study Group et al., 2006); leading to less repeated tests and referrals and a higher sensitivity. In the Netherlands it was found that at age 3 years in 16.6% of the children VA measurements failed and in 15.5% VA was insufficient (Telleman et al., 2019). Since the effectiveness of treatment declines after the age of 5 years (Holmes et al., 2011, Fronius et al., 2014); screening at age 5 years was optimal. However, a screening programme with a single screen at age 5 years is only feasible when a high attendance can be reassured and when repeated screens or diagnosis are not delayed. For a two-screen programme, the model predicted that adding a screen at age 4 years is a good option, especially when the screening is performed by less experienced professionals. There is more time to start the treatment, and a child with amblyopia missed at age 4, can still be detected at age 5 years. More screens, especially at ages younger than 3 years are less optimal. The context in the country should always be taken into account. Besides screening at schools, combining screening with a vaccination booster is a good option, since the attendance is probably high and the overhead costs low. Therefore, the educational and youth healthcare systems will inform whether screening at age 4 or 5 years is optimal.

Strong points of this study are that the model is calibrated on a large vision screening study. In addition, we used utility losses and a life-time horizon, thereby allowing comparison with other healthcare interventions. Furthermore, this is the first cost-effectiveness analysis simulating single or multiple screening at several ages. This was possible by age-specific parameters in the model for prevalence, sensitivity, repeat testing, referral rate and success rate of treatment.

This study also has limitations. The calibration of the model is performed on the Dutch OVAS study that may be less representative for other countries, because of the high acceptability of screening, high coverage of screening and treatment, awareness and good public health infrastructure in the Netherlands. Although the prevalence of amblyopia seems about the same in different countries (Solebo et al., 2015), it is possible that the type or severity of amblyopia differs. In addition, we only modeled VA measurement and not photorefraction, since the impact of photorefraction on the reduction in the development of amblyopia is unknown and the test is rarely used as stand-alone test in Europe (Rostamzad et al., 2020, Horwood et al., 2021).

## 5 Conclusions

Vision screening by measurement of VA can be cost-effective, however, the predictions strongly depend on the assumed utility loss for unilateral visual impairment. Therefore more research is needed to determine the effect of persistent unilateral amblyopia on quality of life. We found for all countries in this study that if only one screen is performed, it can best be done around age 5 years, provided that the attendance is high (for example at schools). Adding a screen at age 4 years is a reasonable option and will increase the QALYs gained by a large amount. Adding more screens generally is less cost-effective.

## Funding

This project has received funding from the European Union’s Horizon 2020 research and innovation programme under grant agreement No. 733352.

## CRediT authorship contribution statement

**Eveline A.M. Heijnsdijk:** Formal analysis, Methodology, Validation, Visualization, Writing – original draft. **Mirjam L. Verkleij:** Methodology, Writing – review & editing. **Jill Carlton:** Funding acquisition, Data curation, Writing – review & editing. **Anna M. Horwood:** Funding acquisition, Data curation. **Maria Fronius:** Funding acquisition, Data curation, Writing – review & editing. **Jan Kik:** Data curation, Writing – review & editing. **Frea Sloot:** Funding acquisition, Data curation, Writing – review & editing. **Cristina Vladutiu:** Funding acquisition, Data curation, Writing – review & editing. **Huibert J. Simonsz:** Funding acquisition, Methodology, Supervision, Writing – review & editing. **Harry J. de Koning:** Funding acquisition, Methodology, Supervision, Writing – review & editing.

## Declaration of Competing Interest

The authors declare that they have no known competing financial interests or personal relationships that could have appeared to influence the work reported in this paper.
