# Supplementary material for: The cost-effectiveness of different visual acuity screening strategies in three European countries: A microsimulation study
Source: Prev Med Rep. 2022 Jun 27;28:101868. doi: 10.1016/j.pmedr.2022.101868 (PMC9253646; doi:10.1016/j.pmedr.2022.101868)
Supplement: Supplementary data 1 [file mmc1.docx]

**Supplement: The cost-effectiveness of different visual acuity screening strategies in three European countries**

**Supplementary Table 1. General parameters in the MISCAN model**

| General parameters | Value | Source |
| --- | --- | --- |
| Incidence of amblyopia at age 7 years | 3.6% | Calibrated on OVAS study^12^ data |
| Probability of:  - refractive error amblyopia  - combined mechanism amblyopia  - strabismic amblyopia  - deprivation amblyopia | 64.3%  7.4%  25.9%  2.4% | Calibrated on OVAS study^12^ data |
| Duration of preclinical state (in years) of:  - refractive error amblyopia  - combined mechanism amblyopia  - strabismic amblyopia  - deprivation amblyopia | 7.0  6.6  5.1  2.5 | Calibrated on OVAS study^12^ data |
| Test sensitivities at age 1, 4 and 6 years  (in between the test sensitivities are interpolated) of:  - refractive error amblyopia  - combined mechanism amblyopia  - strabismic amblyopia  - deprivation amblyopia | 0% - 86% - 96%  45% - 50% - 60%  49% - 56% - 71%  77% - 82% - 87% | Calibrated on OVAS study data and extrapolated for ages above 4 years |
| Treatment success | 75% at age 3 months to 5.5 years, declining to 5% at age 12 | ^6,8^ |

**Supplementary Figure 1:** The estimated cumulative incidence by age and amblyopia type. Blue denotes deprivation amblyopia, red denotes strabismic amblyopia, green denotes combined mechanism amblyopia and grey denotes refractive error amblyopia. The dashed grey line is the refractive error amblyopia that can be detected only when visual acuity is measured. The total cumulative incidence of amblyopia is represented by the black line.

Data of the control arm of the OVAS study were used to calibrate the parameters in the model.

**Supplementary Figure 2:** Cases of amblyopia diagnosed in the control arm of the OVAS study (solid line) and the predicted cases by the MISCAN model by age.

**Supplementary Figure 3:** Screen detected and clinically detected cases of amblyopia by type in the control arm of the OVAS study (observed), versus the model predictions (predicted).

Data of the intervention arm of the OVAS study were used to validate the MISCAN model: The model including the estimated parameters is used to reproduce the intervention arm of the OVAS study.

**Supplementary Figure 4:** Cases of amblyopia diagnosed in the intervention arm of the OVAS study (solid line) and the predicted cases by the model by age.

**Supplementary Figure 5:** Screen detected and clinically detected cases of amblyopia by type in the intervention arm of the OVAS study (observed), versus the model predictions (predicted).

**Supplementary Table 2. Results of all strategies containing one or two screens for the Netherlands. All results are presented for 1,000 children, followed over lifetime.**

|  | 3 years | 4 years | 5 years | 6 years | 3 and 4 years | 3 and 5 years | 3 and 6 years | 4 and 5 years | 4 and 6 years | 5 and 6 years |
| --- | --- | --- | --- | --- | --- | --- | --- | --- | --- | --- |
| Without discount |  |  |  |  |  |  |  |  |  |  |
| Screens (including repeated screens) | 1,070 | 976 | 886 | 866 | 2,031 | 1,943 | 1,925 | 1,846 | 1,829 | 1,738 |
| Additional referrals | 10.1 | 14.4 | 1.5 | 4.7 | 42.1 | 27.2 | 28.4 | 35.5 | 36.4 | 24.5 |
| Cases detected by screening | 17.6 | 19.1 | 17.9 | 16.3 | 25.4 | 25.3 | 24.7 | 24.8 | 24.4 | 22.6 |
| Persistent amblyopia prevented | 4.6 | 5.5 | 5.6 | 4.3 | 6.9 | 7.1 | 6.5 | 7.3 | 6.9 | 6.9 |
| QALYs gained | 3.32 | 3.95 | 4.01 | 2.95 | 4.94 | 5.06 | 4.63 | 5.23 | 4.92 | 4.87 |
| Net costs (screening, diagnosis and treatment) | €15,531 | €17,504 | €15,709 | €16,092 | €27,937 | €26,088 | €26,267 | €26,952 | €27,101 | €24,888 |
| Costs / case detected | €862 | €791 | €724 | €826 | €863 | €770 | €794 | €807 | €826 | €802 |
| Net cost /persistent amblyopia prevented | €3,351 | €3,166 | €2,791 | €3,782 | €4,047 | €3,683 | €4,028 | €3,682 | €3,917 | €3,628 |
| Costs savings | €1,789 | €2,134 | €2,170 | €1,641 | €2,663 | €2,734 | €2,521 | €2,825 | €2,675 | €2,648 |
| With 3.5% discount |  |  |  |  |  |  |  |  |  |  |
| QALYs gained | 0.77 | 0.91 | 0.92 | 0.64 | 1.14 | 1.16 | 1.05 | 1.20 | 1.12 | 1.10 |
| Total net costs | €19,195 | €19,985 | €16,923 | €15,824 | €31,837 | €29,606 | €29,043 | €29,033 | €28,589 | €25,088 |
| ICER* | Dominated | Dominated | €18,399 | Dominated | Dominated | Dominated | Dominated | €42,952 | Dominated | Dominated |

* ICER = the Incremental Cost-Effectiveness ratio: the additional costs divided by the additional QALYs gained, compared with the previous cost-effective strategy.

**Supplementary Table 3. Results of all strategies containing three or more screens for the Netherlands. All results are presented for 1,000 children, followed over lifetime.**

|  | 3, 4 and 5 years | 3, 4 and 6 years | 3, 5 and 6 years | 4, 5 and 6 years | 3, 4, 5 and 6 years |
| --- | --- | --- | --- | --- | --- |
| Without discount |  |  |  |  |  |
| Screens (including repeated screens) | 2,897 | 2,880 | 2,792 | 2,695 | 3,744 |
| Additional referrals | 68.8 | 69.1 | 55.2 | 64.5 | 99.3 |
| Cases detected by screening | 28.2 | 28.1 | 27.5 | 26.5 | 29.1 |
| Persistent amblyopia prevented | 7.8 | 7.6 | 7.6 | 7.8 | 8.0 |
| QALYs gained | 5.56 | 5.42 | 5.44 | 5.52 | 5.73 |
| Net costs (screening, diagnosis and treatment) | €36,092 | €36,185 | €33,954 | €34,519 | €43,231 |
| Costs / case detected | €980 | €986 | €924 | €985 | €1,164 |
| Net cost /persistent amblyopia prevented | €4,631 | €4,755 | €4,441 | €4,454 | €5,379 |
| Costs savings | €3,001 | €2,934 | €2,948 | €2,990 | €3,096 |
| With 3.5% discount |  |  |  |  |  |
| QALYs gained | 1.28 | 1.24 | 1.25 | 1.27 | 1.32 |
| Total net costs | €39,201 | €38,907 | €36,272 | €35,371 | €45,071 |
| ICER | Dominated | Dominated | Dominated | €100,151 | €188,774 |

* ICER = the Incremental Cost-Effectiveness ratio: the additional costs divided by the additional QALYs gained, compared with the previous cost-effective strategy.

**Supplementary Table 4. Screening strategies on the efficiency frontier with corresponding ICERs in the sensitivity analysis. The value of ICERs below € 20,000 is given in brackets.**

| Parameter | ICER < € 20,000 | ICER between  € 20,000 -€ 80,000 | ICER > € 80,000 |
| --- | --- | --- | --- |
| Utility loss unilateral 0.005 |  | 5 years | 4, 5 years  4, 5, 6 years  3, 4, 5, 6 years |
| Utility loss unilateral 0.02 | 5 years (9,262) | 4, 5 years  4, 5, 6 years | 3, 4, 5, 6 years |
| Utility loss bilateral 0.22 | 5 years (17,976) | 4, 5 years | 4, 5, 6 years  3, 4, 5, 6 years |
| Utility loss treatment 0.01 | 5 years (19,548) | 4, 5 years | 4, 5, 6 years  3, 4, 5, 6 years |
| Treatment success 0.65 |  | 5 years  4, 5 years | 4, 5, 6 years  3, 4, 5, 6 years |
| Treatment success 0.85 | 5 years (14,691) | 5, 6 years  4, 5 years  4, 5, 6 years | 3, 4, 5, 6 years |
| Test sensitivity -10% | 5 years (19,043) | 4, 5 years | 4, 5, 6 years  3, 4, 5, 6 years |
| Test sensitivity +10% | 5 years (17,798) | 4, 5 years | 4, 5, 6 years  3, 4, 5 years  3, 4, 5, 6 years |
| Referral -10% | 5 years (17,951) | 4, 5 years | 4, 5, 6 years  3, 4, 5, 6 years |
| Referral +10% | 5 years (18,846) | 4, 5 years | 4, 5, 6 years  3, 4, 5, 6 years |
| 100% attendance | 5 years (18,301) | 4, 5 years | 4, 5, 6 years  3, 4, 5 years  3, 4, 5, 6 years |
| 80% attendance | 5 years (18,458) | 4, 5 years | 4, 5, 6 years  3, 4, 5, 6 years |
| Costs of a screen test -10% | 5 years (17,601) | 4, 5 years | 4, 5, 6 years  3, 4, 5, 6 years |
| Costs of a screen test +10% | 5 years (19,196) | 4, 5 years | 4, 5, 6 years  3, 4, 5, 6 years |
| Costs of diagnosis -10% | 5 years (17,896) | 4, 5 years  4, 5, 6 years | 3, 4, 5, 6 years |
| Costs of diagnosis +10% | 5 years (19,126) | 4, 5 years | 4, 5, 6 years  3, 4, 5, 6 years |
| Costs of treatment -10% | 5 years (10,415) | 4, 5 years | 4, 5, 6 years  3, 4, 5, 6 years |
| Costs of treatment +10% |  | 5 years  4, 5 years | 4, 5, 6 years  3, 4, 5, 6 years |
| Discount of 1% | 5 years (8,004)  4, 5 years (19,477) | 4, 5, 6 years | 3, 4, 5, 6 years |
| Discount of 5% |  | 5 years | 4,5 years  4, 5, 6 years  3, 4, 5, 6 years |

* ICER = the Incremental Cost-Effectiveness ratio: the additional costs divided by the additional QALYs gained, compared with the previous cost-effective strategy.

**Supplementary Figure 6:** The net costs and QALYs gained of the current screening programme in the UK (grey square) and alternative screening strategies. Costs and QALYs are discounted with 3.5%. The black dots are the strategies with one screen, the grey dots with 2 screens, the black triangles with 3 screens and the grey triangle with 4 screens.

**Supplementary Table 5. Results of all strategies on the efficiency frontier for the UK. All results are presented for 1,000 children, followed over lifetime.**

|  | 5 years | 4 and 5 years | 4, 5 and 6 years | 3, 4 and 5 years | 3, 4, 5 and 6 years |
| --- | --- | --- | --- | --- | --- |
| Without discount |  |  |  |  |  |
| Screens (including repeated screens) | 916 | 1,816 | 2,754 | 2,902 | 3,839 |
| Additional referrals | 80.1 | 187.3 | 257.0 | 298.9 | 369.3 |
| Cases detected by screening | 21.6 | 26.5 | 27.4 | 29.4 | 29.8 |
| Persistent amblyopia prevented | 6.8 | 7.8 | 8.0 | 8.1 | 8.2 |
| QALYs gained | 4.81 | 5.51 | 5.66 | 5.70 | 5.78 |
| Net costs (screening, diagnosis and treatment) | €22,384 | €42,343 | €56,001 | €62,804 | €76,402 |
| Costs / case detected | €1,190 | €1,679 | €2,108 | €2,198 | €2,610 |
| Net cost /persistent amblyopia prevented | €3,287 | €5,432 | €6,987 | €7,788 | €9,331 |
| Costs savings | €2,562 | €2,935 | €2,973 | €2,979 | €3,081 |
| With 3.5% discount |  |  |  |  |  |
| QALYs gained | 1.11 | 1.27 | 1.30 | 1.32 | 1.34 |
| Total net costs | €21,170 | €39,417 | €50,426 | €58,589 | €69,512 |
| ICER* | €19,118 | €110,255 | €354,906 | €555,346 | €634,308 |

* ICER = the Incremental Cost-Effectiveness ratio: the additional costs divided by the additional QALYs gained, compared with the previous cost-effective strategy.

**Supplementary Figure 7:** The net costs and QALYs gained of the current screening programme in Romania (grey square) and alternative screening strategies. Costs and QALYs are discounted with 3.5%. The black dots are the strategies with one screen, the grey dots with 2 screens, the black triangles with 3 screens and the grey triangle with 4 screens.

**Supplementary Table 6. Results of all strategies on the efficiency frontier for Romania. All results are presented for 1,000 children, followed over lifetime.**

|  | 5 years | 4 and 5 years | 4, 5 and 6 years | 3, 4, 5 and 6 years |
| --- | --- | --- | --- | --- |
| Without discount |  |  |  |  |
| Screens (including repeated screens) | 758 | 1,507 | 2,267 | 3,143 |
| Additional referrals | 66.3 | 143.4 | 164.8 | 232.2 |
| Cases detected by screening | 14.7 | 21.9 | 25.7 | 28.2 |
| Persistent amblyopia prevented | 4.6 | 6.7 | 7.6 | 8.2 |
| QALYs gained | 2.94 | 4.28 | 4.85 | 5.22 |
| Net costs (screening, diagnosis and treatment) | €16,598 | €26,341 | €32,797 | €38,540 |
| Costs / case detected | €401 | €466 | €511 | €623 |
| Net cost /persistent amblyopia prevented | €3,596 | €3,936 | €4,295 | €4,699 |
| Costs savings | €1,475 | €2,148 | €2,452 | €2,628 |
| With 3.5% discount |  |  |  |  |
| QALYs gained | 0.66 | 0.97 | 1.09 | 1.18 |
| Total net costs | €15,433 | €25,149 | €30,658 | €37,000 |
| ICER* | €23,265 | €31,812 | €47,587 | €70,321 |

* ICER = the Incremental Cost-Effectiveness ratio: the additional costs divided by the additional QALYs gained, compared with the previous cost-effective strategy.
